# Meeting the challenges in conducting research in vulnerable older adults with self-neglect-notes from a field team

**DOI:** 10.3389/fmed.2023.1114895

**Published:** 2023-03-29

**Authors:** Sabrina Pickens, Jason Burnett, Mary Ellen Trail Ross, Erick Jones, Felicia Jefferson

**Affiliations:** ^1^College of Nursing, Texas Woman's University, Denton, TX, United States; ^2^McGovern Medical School, University of Texas Health Science Center at Houston, Houston, TX, United States; ^3^Center for Nursing Research, Cizik School of Nursing, University of Texas Health Science Center at Houston, Houston, TX, United States; ^4^Department of Mechanical Engineering, College of Engineering, University of Nevada, Reno, NV, United States; ^5^Department of Neuroscience, Fort Valley State University, Fort Valley, GA, United States

**Keywords:** self-neglect, aging adults, field notes, vulnerable adult, elder abuse

## Abstract

Gaining a systematic understanding of possible ways to increase the quality and lifespan of older adults experiencing self-neglect has unique challenges. These challenges include identifying self-neglect in the community and navigating levels of cognitive, physical, and/or psychological difficulties in this population that impact recruitment, consent, and accurate data collection. Conducting quality research under some of the environmental self-neglect conditions such as squalor, animal and insect infestations and no utilities can also challenge planned study protocols and study validity. This manuscript presents details of these overarching challenges and some of the workable solutions noted and implemented by research field-team members who have enrolled over 300 adults experiencing self-neglect for various studies. Usual research methodology must overcome these barriers to work to create consciousness about the self-neglect population. The classic series of cases is still a good alternative when describing self-neglect. Considerations for conducting future self-neglect research are presented.

## Introduction

Self-neglect is a form of abuse characterized by the inability or refusal to provide self-care such as bathing, grooming, eating and managing finances (1). This form of self-abuse is associated with acute and chronic medical conditions such as frailty, depression, cognitive disorders, untreated pain, physical impairments, social isolation, and substance abuse (2–4). Despite its inception to the medical literature in the 1960s and being the most common referral to Adult Protective Services (APS), the state agencies charged with investigating abuse, neglect, and exploitation of adults, limited self-neglect research studies have been conducted (5). This is unfortunate as self-neglect exacerbates morbidity (6) and increases mortality risks 2-5 fold compared to older adults not experiencing self-neglect (7, 8). The lack of studies likely reflects the myriad of challenges associated with conducting research in this population.

**Figure 1:**
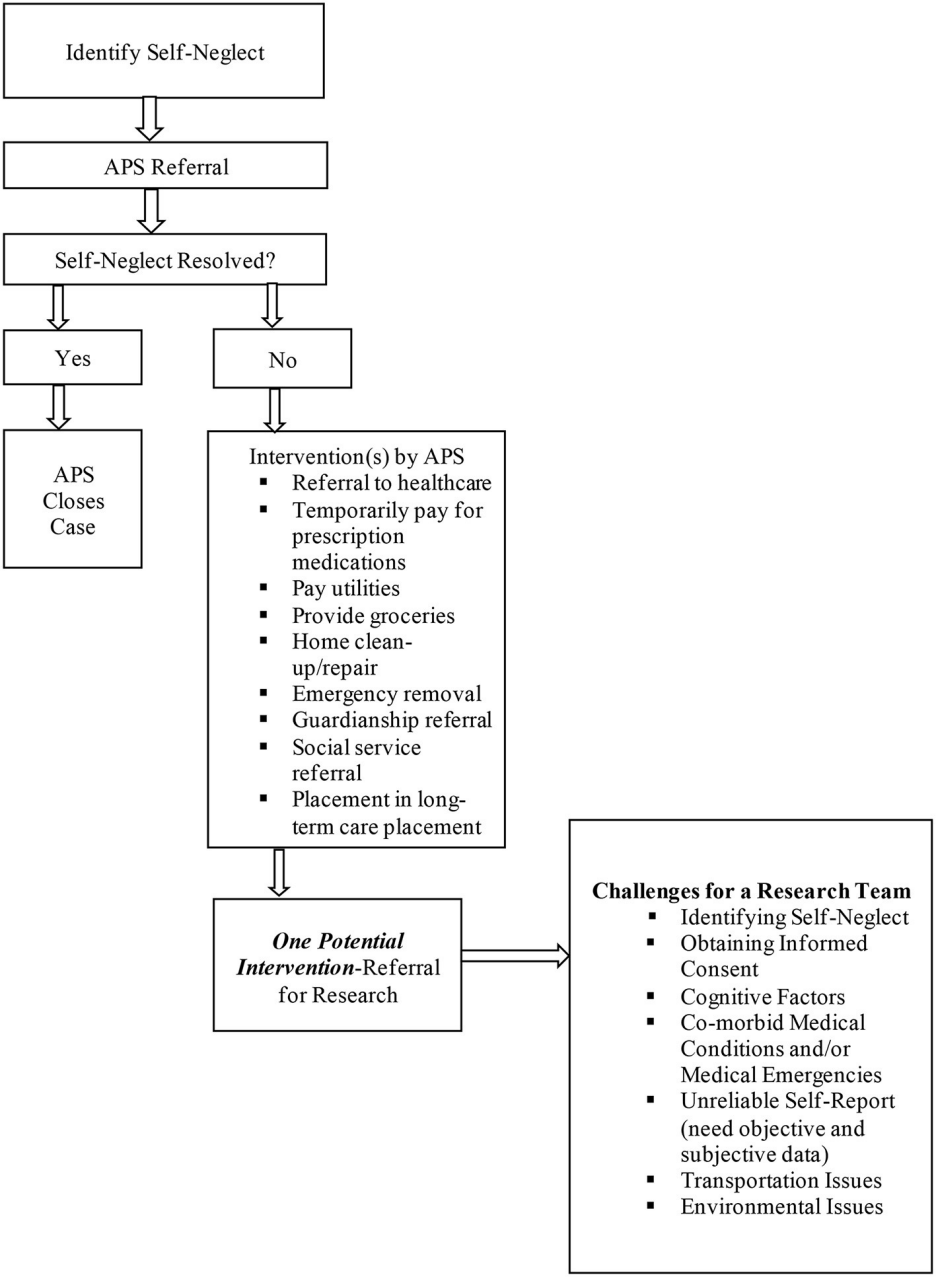
Challenges of recruiting for self-neglect research.

In 1995, the Texas Elder Abuse and Mistreatment Institute (TEAM) was established to provide clinical support to an APS program in Houston, Texas serving large numbers of older adults living in the community with self-neglect. TEAM was originally comprised of clinicians, APS workers, prosecutors, attorneys, community groups, researchers, academics, business entities, and social service agencies. The development of TEAM was to educate healthcare and community service workers, as well as the public about elder abuse. In addition, TEAM conducts research on elder abuse (9). This partnership led to the first National Institutes of Health funded study of self-neglect called the Consortium for Research in Elder Self-neglect of Texas (CREST) in 2005 (HSC-MS-0800052). CREST paved the way for several manuscripts reporting its findings in addition to a variety of subsequent self-neglect studies (2, 5, 10–17) through which the research team (i.e., authors of this manuscript) cataloged challenges and solutions to studying this population. This manuscript describes some of the major research challenges (refer to Figure 1) and workable solutions and provides considerations for future work in this continuously understudied population. Because of the many challenges, we recommend ethics committees and grant agencies to have a particular concession for studies involving self-neglect. Thus, different ethics parameters or flexibility in research protocols should be asked so research in this particular field of study can progress.

## Discussion

### Defining self-neglect

Defining self-neglect is not straightforward. Self-neglect is statutorily defined however, the language varies from state to state and the definition does not commonly differentiate “intentional (being aware)” vs. “unintentional (not being aware)” self-neglect. The federal government describes self-neglect as “the failure to provide for oneself the goods or services that are necessary to avoid physical harm, mental anguish, or mental illness” (18). The National Association of Adult Protective Services Administrators (NAPSA) defines self-neglect as “older adults or adults with disabilities who cannot meet their own essential physical, psychological or social needs, which threatens their health, safety and wellbeing. This includes failure to provide adequate food, clothing, shelter, and health care for one's own needs” (19). These definitions exclude the situation in which a mentally competent older adult, who understands the consequences of his/her decisions, makes a conscious, voluntary and personal decision to engage in acts that may be harmful to her/his health or safety. This distinction is important because the research team found that self-neglect occurs along a continuum ranging from mild to severe. Where a person lands on this subjective continuum is likely associated with some impairment that creates other challenges that are discussed below in greater detail (i.e., cognitive, physical, and psychological impairments). This is supported by a secondary data analysis of statewide APS data, conducted by Burnett et al. (20), which showed that older adults experiencing self-neglect across multiple domains (i.e., global self-neglect) are more likely to have mental health issues.

To help broaden the definition, Pickens et al. (21) recently published a definition for severe self-neglect as “an unawareness to the hazardous and progressive decline in personal, social, physical, mental and/or environmental domains leading to the inability to maintain culture and community standards of acceptable living that threatens one's own safety, health and quality of life”. From a research perspective, adding this definition to a study, helps understand the target population better and perhaps how to navigate associated challenges with recruitment and consent.

### Identifying self-neglect in the community

Older adults who self-neglect are often reclusive and live socially isolated. This makes identification for research difficult. Unlike children, not attending a social event may not raise any flags or lead to social welfare checks. Despite many older adults with self-neglect having undertreated or untreated medical conditions, many of these individuals were being “treated” by a healthcare provider, but not engaging in medical care. For example, despite having medications, adherence was low. It was also discovered that medical orders were not being followed such as lack of wound care, not assessing blood sugars or blood pressure measurements, not using physical aids such as prescription glasses, dentures, canes, walkers or wheelchairs or not wearing incontinence pads or using bedside commodes. Other older adults who self-neglect simply may refuse to seek medical care and thus do not come to the attention of health care providers or systems (22). In fact, a cardinal feature of elder self-neglect is refusal of care. Many of these individuals have advanced medical disease and have not sought healthcare in years, if not decades. Similarly, they neglect social engagements and remain from the many avenues of social scrutiny. When they are recognized as being in danger or in need, they are often reported to state regulatory agencies such as APS which investigate these cases in the older adult's home and seek to support ongoing safety and wellbeing.

Another challenge to identification is the lack of a gold standard measurement of self-neglect. Even APS agencies don not have psychometrically supported measures of self-neglect and rely on non-standardized checklists and/or gestalt feelings for determination. Nevertheless, their determinations are often considered the “gold standard” for research. While some studies have developed self-neglect screening tools, none have been widely studied for validity and reliability and endorsed to replace APS designations. This necessitates creating ongoing trusted partnerships between researchers and social service agencies, such as APS, who can refer clients for study purposes. This is a feasible approach that may be the only way to truly identify these individuals in the community (9).

### Confidentiality and recruitment

Recruiting older adults experiencing self-neglect, even when known to APS, poses its own challenges. Policies and practices around client confidentiality have to be considered and safeguards put in place before releasing information to a research team. First, memorandums of understanding have to be developed between the APS and the research entity to set rules on the transfer, use, and dissemination of client and research data. Specific to recruitment, APS and other community-based agencies are not designed for research and while they may have policies and practices in place to release information to other entities, these safeguarding policies and practices may not cover research or institutions of higher education. Prior to being able to receive names of APS self-neglect clients, the research team had to work with APS administrators, lawyers, and ethicists to develop a recruitment protocol that protected APS clients, first and foremost. The outcome resulted in a protocol in which APS caseworkers collected a signed permission statement, from their client, allowing APS to release the client's name and contact information to affiliated research team members. This method helps protect client confidentiality and gives the client the ability to refuse being contacted without any interruption in APS or other services.

### Obtaining informed consent

Contacting this population is no small challenge even after APS has set the groundwork. Often, it would take multiple calling attempts which could take weeks before having a conversation with the older adult. Even then, they may want to think about the idea of having you visit and request a call back. Being patient and building flexibility into the recruitment and study timeframe for this sort of delay is critical. Often, these individual are skeptical of others and require some rapport building beyond the APS connection.

The initial contact with the APS client serves to provide a summary of the study and schedule a home-visit to obtain informed consent. While at the subject's home, the field team administered the consent form in compliance with to a preset protocol (approved by a medical ethicist panel). This protocol helps protect vulnerable populations such as those who are aged, those with cognitive impairments, and in some cases, those who may have less than a high school education. The research team read the entire consent form to the older adult who followed along with their own copy. The older adult was then required to state the purpose of the study including the risks and benefits, any form of compensation, and the ability to withdraw from the study at any time without disruption in services prior to consenting to the visit. Two signed copies are required and one is left with the signing older adult. Familiarity and rapport with APS and agreement to receive a home-visit by the research team did not guarantee that clients would participate in the study.

### Cognitive factors

Cognitive impairments are common among older adults who experience self-neglect. Impairments can result from dementia, depression, psychiatric illnesses, substance abuse, or simply aging and may hinder informed consent procedures (21) as well as data collection. Short-term memory problems are the most common issues encountered with trying to study this population. If these issues are pronounced and discovered during the initial phone call, further consent may not be pursued. Understanding that short-term memory problems are not tantamount to the inability to effectively provide informed consent, the consent process is designed to ask for affirmation and understanding shortly after certain information is provided (i.e., risks, benefits etc.). In prospective studies, older adults, during follow-up phone calls or visits, may need to be reminded of the study and referred to the copy of the consent that was left behind. Those with a known diagnosis of dementia or who required a legal proxy would be excluded from studies that need to rely on self-report.

In some instances, the older adult may be under the influence of a substance such as alcohol. Instances occurred where an older adult was discovered to be drinking during the interview. This can create artificial cognitive and other impairments that affect the ability to consent and respond to the standardized measures. Under these circumstances, the interview would be rescheduled and if unable, the participant may be dropped from the study.

### Co-morbidities and medical emergencies

Self-neglect is accompanied by multiple co-morbid medical conditions, which may impede data collection (21). For example, functional impairments might not allow the completion of certain tests. Either the participant was too impaired (i.e., bed-bound) to complete certain portions of the battery of tests or a specific portion of the battery of tests was not completed for safety concerns of the subjects. Not uncommon, subjects would become fatigued and depending on the extent of the fatigue either a short rest period would be taken or the visit would be terminated.

More serious challenges arose when medical emergencies were discovered during the visit. Two subjects were found to have life-threatening serum potassium levels. One subject had an extremely elevated blood glucose level (>600 mg/dl) and one subject had a resting heart rate of 28 beats per minute with shortness of breath and fatigue. These conditions required the nurse practitioner, part of the field team, to advise subjects to seek urgent medical care. Initially, each of these subjects refused intervention stating they felt “fine”, however, according to the CREST protocol the APS caseworkers were immediately notified about the life-threatening conditions and they were able to convince the subjects to seek urgent medical care. These conditions and others account for some of the reasons self-report is not always the best approach for studying this population.

### Self-report

Inaccurate self-report was another challenge affecting research study outcomes. This was discovered in two ways. First, during social and medical histories, participants may fail to report certain medical conditions, deny them, or report adherence to medical regimens. This is common in research among older adults not experiencing self-neglect, but it is still important to mention here. Often, after medical review and assessment, they admitted to having medical conditions they previously denied. The research team realized that this may simply be an inability to remember all of their conditions at the time being asked, but then remembered later in the study. As part of the home-based evaluation, the research team reviewed the participant's medication list and performed total pill counts for each medication to obtain objective evidence of adherence to medications. This turned out to be a necessary research practice to get the full picture of their health, but also led to the understanding that medication adherence, polypharmacy and medication regimen complexity were real issues in this population and potential targets for intervention (23, 24).

A small percentage of the participants denied alcohol use although later admitting alcohol use and sometimes abuse when full and empty alcohol containers were discovered hidden from plain eye sight or strewn throughout their home. Using an objective measure of activities of daily living (i.e., basic and instrumental) was also determined to be important because older adults experiencing self-neglect commonly overstate their abilities to take care of certain tasks. This was revealed through discrepancies between what they said and what would be observed in terms of medical adherence or taking care of their environment. It was confirmed through a study comparing self-reported with an objective measure of ADLs (25). These experiences underscore the need for including objective measures, when possible, to study this population.

## Other key challenges

### Transportation

Self-neglect is not commonly studied in clinical settings. These individuals often do not have personal transportation or have not set up access to public transportation for clinical visits. Moreover, many do not want to leave their homes for many reasons including caring for pets. To overcome this major barrier, home-based visits were required and conducted which is a more costly and time-consuming approach. In large metropolitan cities or very rural areas, traveling to enroll and assess one participant can take 4–6 h with 2–3 h for driving.

### Environmental

Aside from the many psycho-social-medical obstacles that must be overcome in conducting home-based research in this population, there are also many environmental factors that impair attempts to collect data. Environmental factors such as in-home gas leaks, extreme environmental clutter, severe structural damage (holes in the floors and walls to the outside), unfavorable heating and lighting conditions, noxious odors and rodent and insect infestations were encountered. Although some of the conditions impose more of a barrier to research than others, all are important concerns that should be expected and addressed when conducting home-based research in the self-neglecting population (22).

For all research, the main concern is safety for both the participants and the researchers. When gas leaks were detected in some of the homes during the course of the study, the gas was turned off at the valve, several windows were opened for ventilation, and proper authorities were notified to resolve the hazardous situation. The danger level increased when the participant was known to be a smoker. Some the subjects lived in extremely cluttered homes which placed constraints on certain tests such as the Physical Performance Test and the 8-foot Get Up and Go test as these types of assessment tools require a certain amount of free space to safely and reliably complete them. Therefore, the research team had to either create a safe area where the tests could be safely performed or to deem the environment too hazardous for the specific tests. This type of environmental factor also poses a threat to data accuracy.

Researchers must also anticipate temperature extremes and reduced lighting conditions when conducting home-based research on elders who self-neglect (22). Prior research indicates that an annual income of five thousand dollars or less is typical of these individuals. Thus, money is often unavailable to repair faulty air conditioners, lighting, heating and other home repairs. As a result, structural damage sometimes led to research being conducted in extremely hot and humid conditions. These also created injury risks for the research team when walking through the homes as holes would be covered with rugs. At times, these vulnerable elders became fatigued and several breaks were required in order to complete the examinations. Poor lighting conditions were commonly encountered, and in some instances, the examination was moved to the front or back porch of the home to gain access to proper lighting.

Vulnerable elders with self-neglect often live in squalor with noxious odors, animal, rodent and insect infestations (22). These conditions were usually a result of multiple animals at the residence and associated with waste products that had not been removed for several years. Some of the structural damage allowed for many animals (usually feline) to enter and leave the home through the floors and walls. On some occasions, the foul odors and insect infestations were pronounced enough to make it necessary to conduct the examinations outside of the home or to request the doors remain open to outside air. Often, the subjects were not bothered by their environment.

When family were in the older adult's life, it was not uncommon for the research team to have to contend with family conflict or hostile paid in-home providers who may request the research team to leave and abandon the study. These requests were generally against the wishes of the older adult and if so, we would consult with the older adult. If this occurred before the consent, the research team contacted APS to determine if the individual requesting the team's departure was a legal guardian capable of making decisions for the subject. If this was not the case then the research team tried to reach a close family member to apprise of the situation and in most cases was granted permission to continue the visit. If no close family member was accessible and the older adult was deemed able to consent, the research team would rely on the older adult's decision. If the team felt that the situation was going to place the older adult at future risk with the provider, they would either decide not to enroll the older adult or ask to come back on another day when the hostile provider was not there.

## Conclusion

Research using primary data collection approaches targeting older adults experiencing self-neglect has been hindered by a myriad of challenges. This manuscript highlights some of the noted challenges and expectations of research in this population and offers practical solutions from a research team with the most experience studying self-neglect in older adults. The challenges can be categorized into groups consisting of (1) identification, recruitment, and consent, (2) cognitive, physical, and emotional, and (3) environmental. Solutions such as partnering with agencies that investigate self-neglect to support identification of this population in the community and developing practical and ethical ways of recruiting and consenting potential participants is paramount. Contending with the different biopsychosocial factors that can interfere with recruitment and consent as well as accurate data collection are also critical to prepare for and address this understudied population. This means trying to ensure that social desirability responses and/or inaccurate responses due to memory impairments are addressed using a mixture of objective and subjective measures. Verification strategies are especially necessary when there are no easy objective assessments of self-reported responses such as medical conditions. Even when the biopsychosocial challenges are handled, the environment or participants home, where self-neglect research is most feasible due to transportation and unwillingness to leave their home, poses data collection challenges that are unparalleled. The research team has to be equipped with strategies for overcoming poor lighting, hazardous structural damage, uncomfortable temperatures, limited spaced to conduct assessments, and hazards such gas leaks, animal, rodent, and insect infestations. There is no single approach to overcoming these conditions, but knowing that they may occur and trying to be prepared before the visit is important to the success of the study.

More studies in this population are needed and this manuscript provides evidence that studies are feasible albeit challenging. Future studies in this population should consider the challenges and solutions presented in this manuscript.

## Data availability statement

The original contributions presented in the study are included in the article/supplementary material, further inquiries can be directed to the corresponding authors.

## Ethics statement

The cross-sectional study involving human participants was approved by the Baylor College of Medicine. The patients/participants provided their written informed consent to participate in the study.

## Author contributions

SP provided substantial contributions to the conception of the work, acquisition and analysis of data for the work, and who agrees to be accountable for all aspects of the work in ensuring that questions related to the accuracy or integrity of any part of the work are appropriately investigated and resolved. FJ, MT, and JB provided substantial contributions to the design of the work, interpretation of the data for the work, and revised it critically for intellectual content. EJ provided substantial contributions in the acquisition and analysis of data for the work. FJ who agrees to be accountable for all aspects of the work in ensuring that questions related to the accuracy or integrity of any part of the work are appropriately investigated and resolved. All authors contributed to the article and approved the submitted version.
